# *Staphylococcus aureus* with an *erm*-mediated constitutive macrolide-lincosamide-streptogramin B resistance phenotype has reduced susceptibility to the new ketolide, solithromycin

**DOI:** 10.1186/s12879-019-3779-8

**Published:** 2019-02-19

**Authors:** Weiming Yao, Guangjian Xu, Duoyun Li, Bing Bai, Hongyan Wang, Hang Cheng, Jinxin Zheng, Xiang Sun, Zhiwei Lin, Qiwen Deng, Zhijian Yu

**Affiliations:** 1 Department of Infectious Diseases and Shenzhen Key Lab for Endogenous Infection, Shenzhen Nanshan Hospital of Shenzhen University, No. 89, Taoyuan Road, Nanshan District, Shenzhen, 518052 China; 2 Key Laboratory of Medical Molecular Virology of Ministries of Education and Health, School of Basic Medical Science and Institutes of Biomedical Sciences, Shanghai Medical College of Fudan University, No.130, Dongan road, Xuhui District, Shanghai, 200032 China

**Keywords:** *Staphylococcus aureus*, Constitutive macrolide-lincosamide-streptogramin B (cMLSB) resistance, Solithromycin

## Abstract

**Background:**

Solithromycin, the fourth generation of ketolides, has been demonstrated potent antibacterial effect against commonly-isolated gram-positive strains. However, *Staphylococcus aureus* (*S. aureus)* strains with a higher solithromycin MIC have already been emerged, the mechanism of which is unknown.

**Methods:**

Antimicrobial susceptibility test was performed on 266 strains of *S. aureus.* The antibiotic resistance phenotype of *erm*-positive strain was determined by D-zone test. Spontaneous mutation frequency analysis was performed to compare the risk levels for solithromycin resistance among different strains. Efflux pumps and mutational analysis of ribosomal fragments as well as *erm(B)* gene domains were detected. Quantitative reverse transcription polymerase chain reaction was conducted to compare the transcriptional expression of the *erm* gene between the constitutive macrolide-lincosamide-streptogramin B (cMLSB)- and inducible MLSB (iMLSB)-phenotypes.

**Results:**

In the *erm-*positive *S. aureus* strains, the minimum inhibitory concentration **(**MIC)_50/90_ of solithromycin (2/> 16 mg/L) was significantly higher than that in the *erm-*negative strains (0.125/0.25 mg/L). Of note, the MIC_50_ value of the strains with iMLSB (0.25 mg/L) was significantly lower than that of the strains with cMLSB (4 mg/L). A comparison among strains demonstrated that the median mutational frequency in isolates with cMLSB (> 1.2 × 10^− 4^) was approximately > 57-fold and > 3333-fold higher than that in iMLSB strains (2.1 × 10^− 6^) and in erythromycin-sensitive strains (3.6 × 10^− 8^), respectively. The differential antibiotic in vitro activity against strains between cMLSB and iMLSB could not be explained by efflux pump carriers or genetic mutations in the test genes. The expression of the *erm* genes in strains with cMLSB did not differ from that in strains with iMLSB.

**Conclusions:**

The reduced susceptibility to solithromycin by *S. aureus* was associated with the cMLSB resistance phenotype mediated by *erm*.

**Electronic supplementary material:**

The online version of this article (10.1186/s12879-019-3779-8) contains supplementary material, which is available to authorized users.

## Background

The emergence and rapid transmission of antibiotic-resistance genes among *Staphylococcus aureus* (*S. aureus*) strains pose serious public health challenges worldwide [1]. The erythromycin ribosome methylase (*erm*) genes encode proteins that methylate adenine residues A2058/2059 in the peptidyl transferase region of 23S rRNA domain V, and are responsible for macrolide, lincosamide, and streptogramin B (MLSB) antibiotic resistance [2]. In some regional reports, the frequency of methicillin-resistant *S. aureus* (MRSA) with macrolide resistance was over 90% while methicillin-sensitive *S. aureus* (MSSA) rose gradually to over 40% [3]. The rapid transmission and broad antibiotic resistance spectrum of *erm* have greatly limited the clinical utility of traditional macrolides such as erythromycin and azithromycin.

The *Erm* gene-mediated resistant strains exhibit two antimicrobial resistance phenotypes, constitutive MLSB (cMLSB) and inducible MLSB (iMLSB). These phenotypes can be distinguished by D-zone test and are due to different molecular regulatory mechanisms. The translation initiation of *erm* in the iMLSB strain is inhibited due to the sequestration of its mRNA ribosome-binding site by the leader peptide as well as dependent on inducers like erythromycin binding to the leader peptide, by which constrains its role and releases the mRNA ribosome-binding site. In contrast, cMLSB strains allow for direct and timely inducer independent translation of transcripts, in that variations in the leader peptide gene sequence abolish its translation [4, 5]. Through this mechanism, the cMLSB phenotype confers resistance to macrolides, lincosamides, and streptogramin B, while the iMLSB phenotype is resistant to macrolides and streptogramin B but sensitive to lincosamides.

The novel agent, solithromycin, has been reported to be effective against erythromycin-resistant strains and have a formidable antibacterial effect with an extensive antibacterial spectrum. The minimum inhibitory concentrations (MICs) of solithromycin in drug-resistant or multi-drug-resistant strains of clinical isolates (methicillin-resistant *S. aureus* and macrolide-lincosamide-streptogramin B-resistant *Streptococcus pneumoniae*) are comparable or generally lower than those of other regularly used antibiotics, such as linezolid or vancomycin. The antibacterial spectrum of solithromycin includes *Haemophilus influenzae*, *Moraxella catarrhalis*, beta-hemolytic streptococci, *Legionella*, *Bordetella pertussis*, *Chlamydophila pneumoniae*, and *S. aureus*. However, *S. aureus* strains with a solithromycin MIC over 32 mg/L have already emerged, the mechanism of which is unknown [6–8].

In order to address this unknown mechanism, we conducted antimicrobial susceptibility testing of solithromycin against *erm*-positive and -negative strains, analysed the differences in MIC distribution among strains, and explored the potential basis of solithromycin resistance.

## Methods

### Bacterial isolates

A total of 266 strains (without repetition) were isolated from patients in the Nanshan District People’s Hospital of Shenzhen, China from 2013 to 2016. These strains were recovered from respiratory tract secretions, blood, pus, and wound fluid. Strains were identified using the BD Phoenix™-100 Automated Microbiology System (Bd-bio, NJ, USA) according to the manufacturer’s instructions. The presence of the *erm*, *mecA*, and *femB* genes was confirmed by polymerase chain reaction (PCR) as previously described [9, 10]. The verified strains were stored at − 80 °C in Tryptic Soy Broth (TSB) containing 40% glycerol. All procedures involving human participants were performed in accordance with the ethical standards of the Shenzhen University School of Medicine and the 1964 Helsinki declaration and its later amendments. For this type of study, formal consent is not required.

### D-shaped zone of inhibition

All 236 strains carrying the *erm* genes were tested by the previously described disc diffusion method for phenotypic identification [11]. The erythromycin and clindamycin susceptibility test discs were purchased from Sigma Aldrich (St. Louis, MO, USA), and the erythromycin and solithromycin powders were purchased from Cempra Pharmaceuticals (Chapel Hill, USA). After 16 to 18 h of incubation, the *D-zone* test results were assessed by transmitted or reflected light and interpreted according to the Clinical and Laboratory Standards Institute (CLSI) guidelines [12].

### Antimicrobial susceptibility testing

Antimicrobial susceptibility test of these strains was performed by the agar dilution method, following the guidelines of the CLSI. The minimum inhibitory concentration (MIC) was defined as the lowest concentration of antibiotic that completely inhibited growth of the organism in the agar plate as detected by the unaided eye [12]. The MICs for erythromycin and solithromycin were determined. MIC_50_ and MIC_90_ values were defined as the lowest concentration of the antibiotic at which 50 and 90% of the isolates were inhibited, respectively. CLSI breakpoints were used for MIC interpretation.

### Spontaneous mutation frequency

The protocol for how to detect the spontaneous mutation frequency of solithromycin resistance is referred to a previous report [13], in an effort to compare the risk levels between the *erm-*positive and -negative groups as well as the iMLSB and cMLSB groups. Median frequencies were calculated for each group and were used to infer frequency ratios among different groups. Representatives of putative mutant colonies for each plate and group were reassessed for solithromycin MIC determining as described above.

### Amplification and sequencing of efflux pump, ribosomal, and erm genes

The roles of efflux pumps, drug binding sites and *erm* gene mutations in the transformation of *S. aureus* from solithromycin susceptibility to resistance were assessed. To do so, 60 *erm(B)-*positive representative strains were selected and divided into two groups, one with solithromycin MIC values ≤1 mg/L and the other with MIC values ≥4 mg/L. PCR amplification and sequencing were employed to screen for efflux and gene mutations.

Total DNA from all isolates was extracted and purified with the DNeasy Blood and Tissue Kit (QIAGEN China Co., Shanghai, China) according to the manufacturer’s protocol for Gram-positive bacteria. Efflux pumps, *MsrA/B* and *MefE/A,* were detected by PCR. Macrolide binding position mutations for the six copies of *S. aureus rrn* operons (including 5S, 16S, and 23S rRNA genes) and ribosomal proteins [L3 (*rplC*), L4 (*rplD*), and L22 (*rplV*)] were amplified using previously described primers and cycling parameters [13, 14]. The *ermB* gene coding and leader peptide regions were amplified using primers ermBF/ermBR and ermBLF/ermBLR, respectively (Additional file 1: Table S1). PCR products were sequenced (BGI, Shenzhen, China) with specific primers (Additional file 1: Table S1).

### Quantitative reverse transcription-polymerase chain reaction (qRT-PCR)

To explore the impact of transcriptional expression of *erm(A)*, *erm(B)*, and *erm(C)* on solithromycin MIC values, qRT-PCR was performed for iMLSB strains with solithromycin MICs ≤1 mg/L and cMLSB strains with solithromycin MICs ≥4 mg/L.

Total bacterial RNA was extracted using the RNeasyH Mini Kit and reverse transcribed into cDNA using iScript reverse transcriptase (Bio-Rad, Hercules, CA, USA) according to the manufacturer’s instructions. Subsequently, qRT-PCRs were performed using SYBR green PCR reagents (Premix EX TaqTM, Takara Biotechnology, Dalian, China) with the Mastercycler realplex system (Eppendorf AG, Hamburg, Germany). As a reference gene, 16S rRNA was used to normalize transcriptional levels. All qRT-PCRs were carried out in triplicate with at least three independent RNA samples. The primers are listed in Additional file 1: Table S1.

### Statistical analysis

The data were not normally distributed so median mutational frequencies were calculated for each group. Statistical analysis was carried out using SPSS for windows (version 19.0; SPSS, Chicago, IL, USA). The difference in positive percentages (%) between groups was compared by the chi-square test. MIC values for different bacterial groups were compared using the Kruskal-Wallis test. A *P* value of less than 0.05 was considered statistically significant.

## Results

### Percentages of iMLSB and cMLSB strains

**Fig. 1:**
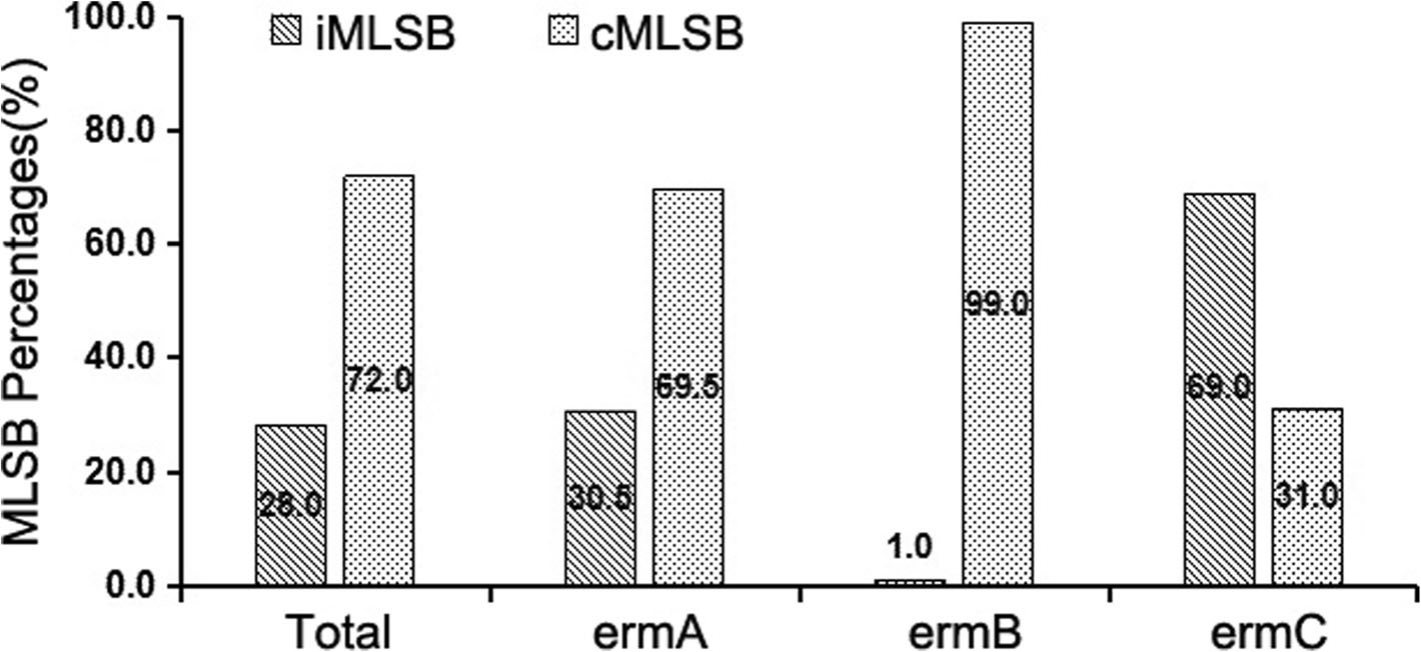
The percentages of MLSB phenotypes in *erm*-positive strains. The specific constitutional rates of iMLSB and cMLSB phenotypes in *erm(A)/(B)/(C)*-positive strains. MLSB phenotype was determined by D-zone testing

Among the 266 isolates, the number of *erm(A)-*, *erm(B)-*, *erm(C)-*positive, *erm-*negative, MRSA, and MSSA strains were 82, 96, 58, 30, 148, and 88, respectively. Among the 236 *erm-*positive strains assessed by the *D-zone* test, the percentage of iMLSB phenotype in *erm(B)*-positive strains was the lowest, comprising 1% of the strains (1/96), the *erm(A)*-positive strains comprised 30.5% (25/82), and the *erm(C)*-positive strains comprised 69.0% of the strains (40/58). In contrast, the percentage of cMLSB phenotype in *erm(B)*-positive strains was the highest, comprising 99.0% (95/96), the *erm(A)*-positive strains comprised 69.5% (57/82), and the *erm(C)*-positive strains comprised 31.0% of the strains (18/58) (Fig. 1).

### In vitro antimicrobial activity of solithromycin for the clinical *S. aureus* isolates

**Fig. 2:**
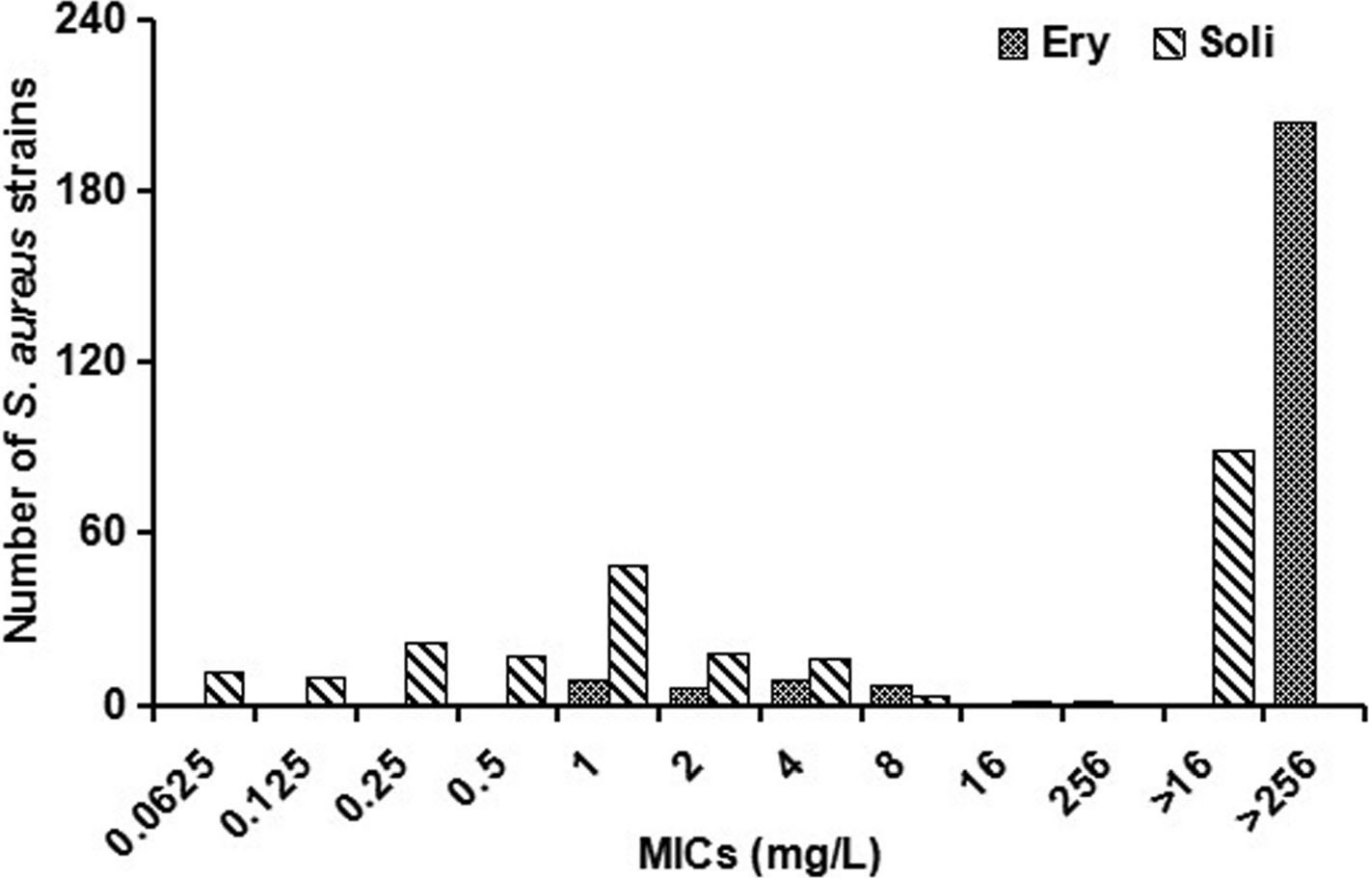
The number of strains with each MIC of erythromycin and solithromycin for the *erm(A)/(B)/(C)*-positive strains. The number of isolates with each MIC of erythromycin and solithromycin was counted after antimicrobial susceptibility testing, by which differences in antibacterial effect were compared between erythromycin and solithromycin. MIC was determined by the agar dilution method according to the guidelines of the CLSI

By the antimicrobial susceptibility test, 53.8% (127/236) of the *erm*-positive strains were inhibited with an MIC of 2 mg/L, 46.2% (109/236) of these strains exhibited resistance with MICs exceeding 4 mg/L. The *erm*-positive strains had a MIC_50_ of 2 mg/L and a MIC_90_ of > 16 mg/L (Fig. 2 and Table 1).

**Table 1:** Antibacterial activity of erythromycin and solithromycin against *S. aureus* isolates as well as the resistant rates of solithromycin in each group

Types (No. of isolates)	Erythromycin MICs			Solithromycin MICs			Solithromycin MICs		*P* values
	(mg/L)			(mg/L)			(mg/L)		
	Range	MIC_50_	MIC_90_	Range	MIC_50_	MIC_90_	≤2	≥4	
Total (236)	1‐>256	>256	>256	0.0625‐>16	2	>16	127 (53.8%)	109 (46.2%)	<0.01
ermA (82)	1‐>256	>256	>256	0.0625‐>16	1	>16	65 (79.3%)	17 (20.7%)	
ermB (96)	1‐>256	>256	>256	0.125‐>16	8	>16	32 (33.3%)	64 (66.7%)	
ermC (58)	1‐>256	>256	>256	0.125‐>16	2	>16	30 (51.7%)	28 (48.3%)	
MSSA (88)	1‐>256	>256	>256	0.0625‐>16	2	>16	48 (54.6%)	40 (45.4%)	0.86
MRSA (148)	1‐>256	>256	>256	0.0625‐>16	2	>16	79 (53.4%)	69 (46.6%)	
iMLSB (66)	1‐>256	>256	>256	0.0625‐>16	0.25	>16	54 (81.8%)	12 (18.2%)	<0.01
cMLSB (170)	1‐>256	>256	>256	0.0625‐>16	4	>16	73 (42.9%)	97 (57.1%)	
ERY^S^ (30)	1‐1	1	1	0.125–0.25	0.125	0.25			

**Fig. 3:**
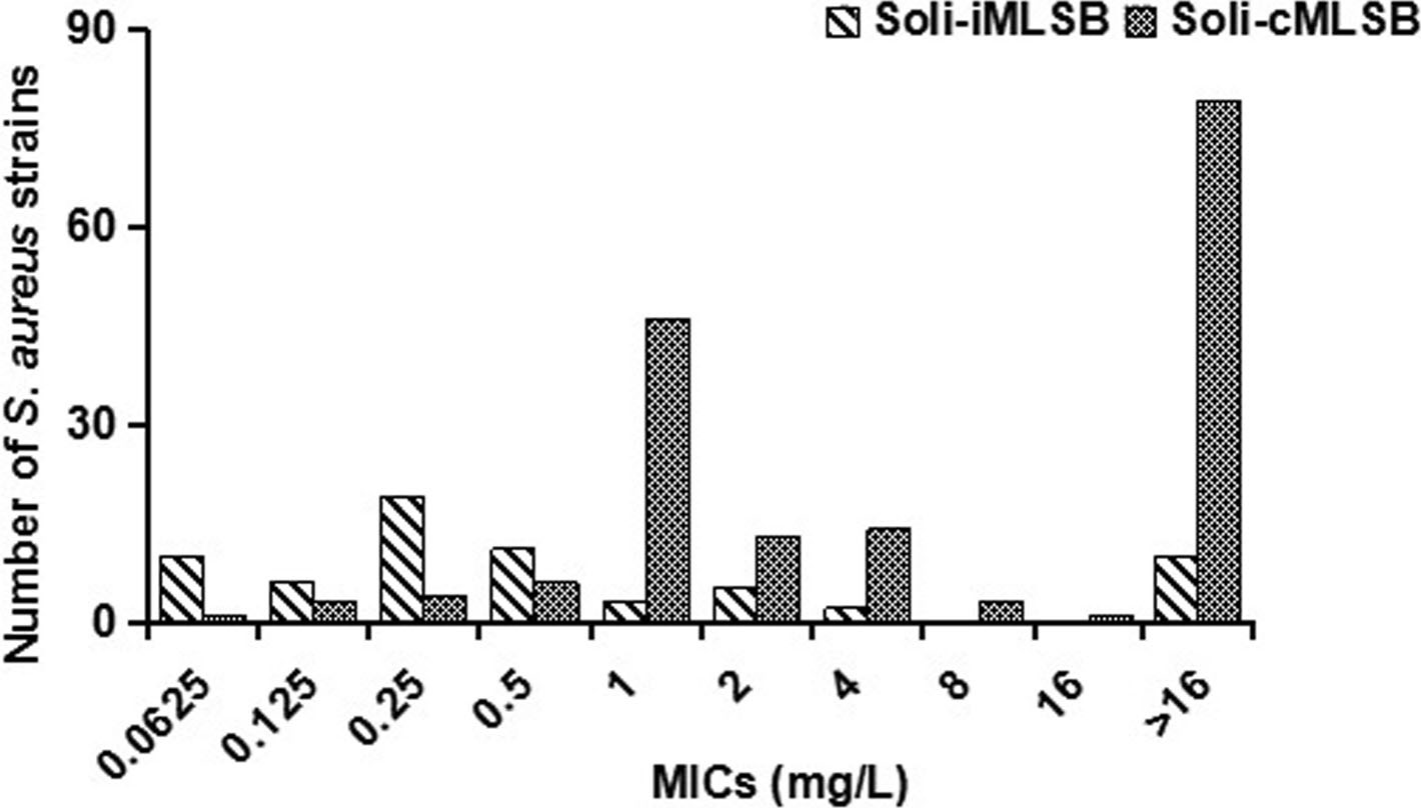
The number of strains with each solithromycin MIC for the iMLSB and cMLSB strains. The number of isolates with each solithromycin MIC was counted separately for *erm*-mediated different MLSB phenotypes, by which the difference in solithromycin antibacterial effect between iMLSB- and cMLSB-phenotypic strains was compared. MIC was determined by the agar dilution method according to the guidelines of the CLSI

The cMLSB phenotype appeared to be associated with reduced solithromycin susceptibility*.* The MIC_50_ of cMLSB strains (4 mg/L) dramatically surpassed that of iMLSB strains (0.25 mg/L). The resistance rate of cMLSB strains (57.1%, 97/170) was significantly higher than that of iMLSB strains (18.2%, 12/66). The MIC_50_ of *erm(B)*-positive strains (8 mg/L) was greater than that of *erm(A)*- and *erm(C)*-positive strains (1 and 2 mg/L, respectively). Therefore, the percentages of the cMLSB phenotype in *erm(B)*-positive strains (99.0%) exceeded those in *erm(A)*- and *erm(C)*-positive strains (69.5 and 31.0%, respectively) (Table 1 and Fig. 3).

### Spontaneous mutation frequencies of erm-positive and -negative strains

The *erm*-mediated cMLSB strains had the highest spontaneous mutation frequency, followed by the *erm*-mediated iMLSB strains and the *erm*-negative strains. The median mutation frequency of the cMLSB strains (> 1.2 × 10^− 4^) was > 57-fold and > 3333-fold higher than that of the iMLSB strains (2.1 × 10^− 6^) and the *erm*-negative strains (3.6 × 10^− 8^), respectively. The time required for the cMLSB strains to exhibit mutants was shorter than that required for the iMLSB strains and the *erm*-negative strains (Table 2). For each group, putative mutant colonies appearing on 2 × MIC solithromycin Müller-Hinton agar (MHA) plates, had reduced susceptibility to solithromycin (Additional file 1: Table S2).

**Table 2:** Spontaneous mutational frequencies of strains with different categories of *erm* and *erm* negative strains toward solithromycin

Types (No. of isolates tested)	Range of mutational frequencies (Median)	Hours of subculture to selection (h)	Erm^+^/Erm^-^ ratio^a^ (Frequencies)
iMLSB in total	1.3 × 10^-5^ to 4.2 × 10^-8^(2.1 × 10^-6^)	40 to 88	59
ErmA^+^(11)	1.3 × 10^-5^ to 4.2 × 10^-8^ (2.7 × 10^-6^)	64 to 88	75
ErmC^+^(5)	2.7 × 10^-6^ to 6.3 × 10^-8^(7.8 × 10^-7^)	40	21
cMLSB in total	>3.9 × 10^-4^ to >4.2 × 10^-5^ (>1.2 × 10^-4^)	40	>3333
ErmA^+^(5)	>3.9 × 10^-4^ to >8.7 × 10^-5^(>3.2 × 10^-4^)	40	>9048
ErmB^+^(12)	>1.6 × 10^-4^ to >4.2 × 10^-5^(>1.2 × 10^-4^)	40	>3277
Erm^-^(26)	4.6 × 10^-7^ to <9.8 × 10^-9^ (3.6 × 10^-8^)	40 to >88	

^a^means the ratio between *erm*-positive strains and *erm*-negative strains in rising folds

### Efflux detection and mutation analysis of macrolide-targeted sites and erm(B) gene

After PCR amplification and sequencing, the efflux pumps were detected negative. Meanwhile, among the 60 representatives, no site mutations, as previously reported to have a definite relationship with macrolide antibiotics resistance, were founded either in the ribosomal genes or *erm(B)*. Some mutations detected may be just nonsense or randomized events during cell replication because they were out of any laws on their distributions (Additional file 2: Table S3).

### Expression of erm(A), erm(B), and erm(C) genes by iMLSB and cMLSB strains

To our surprise, after qRT-PCR of these genes, expression of the *erm*-positive strains with the cMLSB phenotype appeared to be slightly lower than that of the strains with the iMLSB phenotype. In comparison with the iMLSB phenotype, the relative expression of *erm(A), erm(B)*, *erm(C)* in strains with the cMLSB phenotype was 0.23-, 0.54-, and 0.38-fold, respectively (Additional file 3: Figure S1).

## Discussion

Limited data shows that solithromycin has a more potent antimicrobial activity against a variety of bacteria than traditional and novel marolides such as erythromycin and telithromycin. According to previous reports, solithromycin MIC_50/90_ values were 0.008/0.12 mg/L for *S. pneumoniae*, 0.06/0.12 mg/L for *Moraxella catarrhalis*, 0.015/0.03 mg/L for beta-hemolytic streptococci*,* 1/2 mg/L for *Haemophilus influenzae,* 0.06/0.06 mg/L for MSSA, and 0.06/> 32 mg/L for MRSA [7]. Solithromycin exhibits different in vitro antimicrobial activity against *S. aureus*. Assessing this difference is critical because the effect of solithromycin on *S. aureus* in China is unclear. In this study, we found several specific features of solithromycin susceptibility in *S. aureus* strains from China. First, the solithromycin MIC_50/90_ values for the *erm*-positive and -negative *S. aureus* strains were 2/> 16 mg/L and 0.125/0.25 mg/L, respectively, indicating that the solithromycin resistant strains were mainly *erm*-positive. Second, MSSA and MRSA have similar solithromycin MIC_50/90_ values with no significant difference (2/> 16 mg/L), which is different from a previous investigation, in which solithromycin resistance was predominate in MRSA strains [7]. Third, the MIC_50_ values for the iMLSB strains were dramatically lower than those of the cMLSB strains, suggesting that the *erm*-mediated cMLSB phenotype increases solithromycin MICs and is a signature pattern for solithromycin resistance. To the best of our knowledge, no other reports have demonstrated that the cMLSB phenotype in *S. aureus* predicts solithromycin resistance. Although strains with the cMLSB phenotype primarily exhibited solithromycin resistance, a few cMLSB strains did show low solithromycin MIC values. The majority of *S. aureus* strains with the iMLSB phenotype were sensitive to solithromycin, but whether possession of the *erm* gene increases the risk for solithromycin resistance under antibiotics pressure needs to be determined.

The spontaneous mutation frequency is a simple and practical method to evaluate the resistance risk during antibiotic pressure [15] and is used to assess the effect of cMLSB on solithromycin resistance in *S. aureus* and the resistance risk for the iMLSB phenotype. The data demonstrate that harboring *erm* genes predicts the risk for solithromycin resistance to antibiotic stress, as solithromycin-sensitive *S. aureus* with cMLSB phenotype is at greater risk than with iMLSB phenotype, which were at greater risk than erythromycin-sensitive *S. aureus*. This conclusion is consistent with previous findings reported by Pamela McGhee et al., who demonstrated that the degree of solithromycin resistance in *erm(B)*-positive *S. pneumoniae* and *Streptococcus pyogenes* strains was greater than that in erythromycin-sensitive counterparts. However, this report did not demonstrate that *erm* gene mediated cMLSB was a signature of solithromycin resistance [16]. In summary, the solithromycin-sensitive strains of *S. aureus* with cMLSB have an increased risk of resistance, which is far higher than that of strains with iMLSB or with erythromycin-sensitivity. It is well known that pharmacological effects are influenced by a number of factors including antimicrobial susceptibility and desirable pharmacokinetic and pharmacodynamic parameters, such as high bioavailability. Therefore, further evaluation is necessary to determine the clinical significance of solithromycin-sensitive strains with the iMLSB phenotype (and potentially high resistance mutation frequency) during solithromycin antibiotic pressure.

Like the first approved ketolide antibiotic, ie, telithromycin, several possible mechanisms may explain resistance: (1) *erm* aberrance such as deletions and mutations in its promoter region, leader sequences, and coding sequences [17–19]; (2) mutations in the 23S rRNA domains II or V including A138G, C724T, U754A, A2058G, and C2611U [20, 21]; (3) variations in riboproteins L4 or L22 containing insertions, deletions, or mutations of amino acids [22–24]; (4) over-expression of active efflux pumps like *mef* [25]. In order to determine whether the cMLSB phenotype is a major determinant of solithromycin resistance, other mechanisms of macrolide resistance are ruled out. First, the genetic mutations at drug binding sites, including the 23S rRNA gene and the genes encoding the ribosomal proteins L3 (*rplC*), L4 (*rplD*), and L22 (*rplV*), showed no mutation at the target sites, indicating that the target-site mutations were unlikely to be involved in *erm* gene-mediated resistance. Second, the participation of the efflux pumps was also excluded from the solithromycin resistance of *S. aureus* with the cMLSB phenotype. With these exclusions, over-expression and/or genetic polymorphisms of *erm* genes were candidates for the underlying mechanism(s) for solithromycin resistance in *S. aureus* with the cMLSB phenotype. Whereas, no genetic polymorphism in *erm* genes was found to explain the various in vitro antimicrobial activities of solithromycin with cMLSB and iMLSB. Moreover, in this study, the transcriptional expression of the *erm* genes in the cMLSB strains with solithromycin resistance appeared to be even slightly lower than that in iMLSB strains with solithromycin sensitivity. In view of previous reports, erythromycin-induced *erm(B)* expression was regulated at the translational but not the transcriptional level by a translational attenuation/arrest mechanism [5, 26, 27]. Thus, differential *erm* gene expression can still explain the results because *erm* gene expression in the cMLSB strains was independent of the inducers and had a relatively higher protein level than in the iMLSB strains. In addition, the increased expression of *erm* proteins may increase A2058-methylation in rRNA molecules, which is positively correlated with the up-regulation of ketolide MICs [28]. Consequently, cMLSB *S. aureus* strains with larger percentages of A2058-methylation had lower susceptibility to solithromycin than their counterparts iMLSB strains, which became self-evident. For the strains with solithromycin MICs of more than 16 mg/L in iMLSB isolates and the strains with solithromycin MICs of less than 1 mg/L in cMLSB isolates, the mechanism may be the incomplete methylation of A2058, regardless of the phenotype, but different degree of methylation among bacterial strains [28].

## Conclusions

In summary, solithromycin was found to have desirable antimicrobial activity against *S. aureus*, similar to previous reports. However, its antibacterial effect is partially counteracted by the erm-mediated cMLSB resistance phenotype. These findings will benefit the clinical application and management of bacterial infections using solithromycin.

## Additional files

Additional file 1: **Table S1.** Primers used to amplify, sequence and quantify *S. aureus* efflux pumps, 23 s rRNA, ribosomal proteins and *erm* genes throughout the whole study. **Table S2.** Antibacterial activity of solithromycin against *S. aureus* strains appeared on solithromycin containing at 2 times the MIC plates. (DOC 61 kb)

Additional file 2: **Table S3.** 23S rRNA II/V domains, ribosomal proteins, ermB gene mutations of the selected isolates. (XLS 89 kb)

Additional file 3: **Figure S1.** The relative RNA expression levels of *erm(A)/(B)/(C)* genes in different MLSB phenotypic groups. The expression level of RNA transcribed by *erm* genes in different phenotypic groups was measured by quantitative reverse transcription-polymerase chain reaction. The method used to calculate and compare the relative expression levels of RNA in cMLSB strains was 2^-ΔΔCT^; the expression of RNA in iMLSB strains was used as a reference. (TIF 1075 kb)

## Abbreviations

CLSI: Clinical and Laboratory Standards Institute
cMLSB: Constitutive macrolide-lincosamide-streptogramin B
erm: Erythromycin ribosome methylase
iMLSB: Inducible macrolide-lincosamide-streptogramin B
MICs: Minimum inhibitory concentrations
MRSA: Methicillin resistant Staphylococcus aureus
MSSA: Methicillin sensitive Staphylococcus aureus
