# Supplementary material for: Staphylococcus aureus with an erm-mediated constitutive macrolide-lincosamide-streptogramin B resistance phenotype has reduced susceptibility to the new ketolide, solithromycin
Source: BMC Infect Dis. 2019 Feb 19;19:175. doi: 10.1186/s12879-019-3779-8 (PMC6381629; doi:10.1186/s12879-019-3779-8)
Supplement: Supplementary file 1 — Table S1. Primers used to amplify, sequence and quantify S. aureus efflux pumps, 23 s rRNA, ribosomal proteins and erm genes throughout the whole study. Table S2. Antibacterial activity of solithromycin against S. aureus strains appeared on solithromycin containing at 2 times the MIC plates. (DOC 61 kb) [file 12879_2019_3779_MOESM1_ESM.doc]

**Table S1. Primers used to amplify, sequence and quantify *S. aureus*****efflux pumps, 23s rRNA, ribosomal proteins and *erm*** genes throughout the whole study.

| **Primer** | **Sequence (5′ to 3′)** | **References** |
| --- | --- | --- |
| **rrn1F** | GCGGTGTTTTGAGAGATTATTTA | 14 |
| **rrn1R** | GCTTCATGATATACGCTTCCTTT | 14 |
| **rrn2F** | GCAGACGCACAGGACTTA | 14 |
| **rrn2R** | GATACCGTCTTACTGCTCTTCTC | 14 |
| **rrn3F** | AGGCCGGCAATATGTAAG | 14 |
| **rrn3R** | GTCGTCAAACGGCACTAATA | 14 |
| **rrn4F** | TGTGGACGGTGCATCTGTAG | 14 |
| **rrn4R** | ATCACCCGCTCCATAGATAAT | 14 |
| **rrn5F** | GCCGATAGCTCTACCACTG | 14 |
| **rrn5R** | AGGTGCGATGGCAAAACA | 14 |
| **rrn6F** | GAAAGGCGTAACGATTTGGG | 14 |
| **rrn6R** | CGTTGACATATTGTCATTCAG | 14 |
| **RibosomalF** | ATGGGCTTAAACTTACCATC | This study |
| **RibosomalR** | AATCACGGATAATACCAACACG | This study |
| **ErmBF*** | ATGAACAAAAATATAAAA | This study |
| **ErmBR** | TTATTTCCTCCCGTTAAA | This study |
| **ErmBLF*** | ATGTTGGTATTCCAAATG | This study |
| **ErmBLR** | TTATTTCCTCCCGTTAAA | This study |
| **MsrAF** | AAGTTATATCATGAATAGATTGTCCTGTT | 15 |
| **MsrAR** | GGCACAATAAGAGTGTTTAAAGG | 15 |
| **MsrBF** | AAGTTATATCATGAATAGATTGTCCTGTT | 15 |
| **MsrBR** | TATGATATCCATAATAATTATCCAATC | 15 |
| **MefE/AF** | AGTATCATTAATCACTAGTGC | 15 |
| **MefE/AR** | TTCTTCTGGTACTAAAAGTGG | 15 |
| **ermA-F^#^** | ATCTGCAACGAGCTTTGGGT | This study |
| **ermA-R^#^** | ATGCTTCAAAGCCTGTCGGA | This study |
| **ermB-F^#^** | TGAAAGCCATGCGTCTGACA | This study |
| **ermB-R^#^** | GCAACCCTAGTGTTCGGTGA | This study |
| **ermC-F^#^** | CAAAGCGCTCATTGGCGTTA | This study |
| **ermC-R^#^** | TCCCGCATGTTTTAAGGATTTGT | This study |
| **16S-rRNAF^#^** | TCGTGTCGTGAGATGTTGGG | This study |
| **16S-rRNAR^#^** | GTTTGTCACCGGCAGTCAAC | This study |
| **V domain F*** | GCGGTCGCCTCCTAAAAG | 14 |
| **rplCF*** | ATGGGCTTAAACTTACCATC | 14 |
| **rplDF*** | AAAAGGTTTAGTAGAAATCAG | 14 |
| **rplVF*** | GTACATTCAAAGGACACGTTG | 14 |

*** Primers for sequencing; ^#^ Primers used in qRT-PCR.**

**Table S2. Antibacterial activity of solithromycin against *S. aureus* strains appeared on solithromycin containing at 2 times the MIC plates.**

| **Types (No. of isolates tested)** | **Range of MICs in mutant strains (MIC_50_)** | **Range of MICs in wild-type strains (MIC_50_)** | **Rising folds of MICs (mutant /wild-type MIC_50_)** | ***Erm^+^*/*Erm^-^* ratio* (rising folds)** |
| --- | --- | --- | --- | --- |
| **iMLSB in total** | 0.06->16(>16) | 0.06-0.25(0.06) | >267 | >67 |
| **ErmA^+^(18)** | 0.06->16 (1) | 0.06-0.06(0.06) | 17 | 4 |
| **ErmC^+^(16)** | 4->16 (>16) | 0.25-0.25(0.25) | >64 | >16 |
| **cMLSB in total** | 1->16(>16) | 1-1(1) | >16 | >4 |
| **ErmA^+^(15)** | >16->16(>16) | 1-1(1) | >16 | >4 |
| **ErmB^+^(15)** | 1->16(>16) | 1-1(1) | >16 | >4 |
| **Erm^-^(17)** | 0.25-4 (0.5) | 0.125-0.125(0.125) | 4 |  |

*** means the ratio between *erm-*positive strains and *erm-*negative strains in rising folds**
