# Supplementary figures and images for: Staphylococcus aureus with an erm-mediated constitutive macrolide-lincosamide-streptogramin B resistance phenotype has reduced susceptibility to the new ketolide, solithromycin
Source: BMC Infect Dis. 2019 Feb 19;19:175. doi: 10.1186/s12879-019-3779-8 (PMC6381629; doi:10.1186/s12879-019-3779-8)

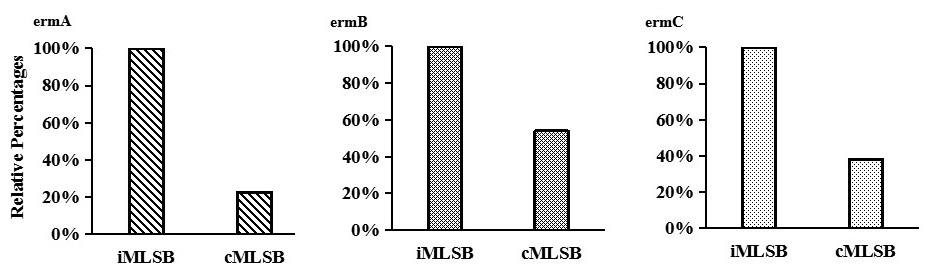

Supplement: Supplementary file 3 — Figure S1. The relative RNA expression levels of erm(A)/(B)/(C) genes in different MLSB phenotypic groups. The expression level of RNA transcribed by erm genes in different phenotypic groups was measured by quantitative reverse transcription-polymerase chain reaction. The method used to calculate and compare the relative expression levels of RNA in cMLSB strains was 2-ΔΔCT; the expression of RNA in iMLSB strains was used as a reference. (TIF 1075 kb) [file 12879_2019_3779_MOESM3_ESM.tif]
